# Low-profile metasurface-backed wideband antenna array for mm-wave applications

**DOI:** 10.1038/s41598-026-37435-9

**Published:** 2026-03-07

**Authors:** Saad Hassan Kiani, Umair Rafique, Nosherwan Shoaib, Muhammad Inam Abbasi, Hela Elmannai, Mohd. Imran Ibrahim, Mariana Dalarsson

**Affiliations:** 1 Fakulti Teknologi Dan Kejuruteraan Elektronik Dan Komputer, Centre for Telecommunication Research and Innovation (CeTRI), Universiti Teknikal Malaysia Melaka, Melaka, Malaysia; 2 Faculty of Information Technology and Electrical Engineering Centre for Wireless Communications, University of Oulu, 90570 Oulu, Finland; 3 School of Electrical Engineering and Computer Science (SEECS) , National University of Sciences and Technology (NUST) , Islamabad, 44000 Pakistan; 4 Department of Information Technology, College of Computer and Information Sciences, Princess Nourah bint Abdulrahman University, P.O. Box 84428, 11671 Riyadh, Saudi Arabia; 5 School of Electrical Engineering and Computer Science , KTH Royal Institute of Technology , 100-44 Stockholm, Sweden

**Keywords:** 5G, Antenna array, High-gain, Metasurface reflector, Mm-wave, Wide bandwidth, Engineering, Physics

## Abstract

We propose a design of 1 × 4 planar antenna array for wideband millimeter-wave (mm-wave) applications, integrated with a metasurface reflector. The single radiating element of the array is composed of a modified ring resonator, designed by combining two rings of different radii. This leads to a compact antenna with dimensions reducing the overall size of the array. For the excitation of array elements, a broadband feeding network is designed, while the wideband characteristics are achieved using a partial ground plane loaded with a square notch. For high gain and improved radiation characteristics, an array of 3 × 10 metasurface unit cells are built and placed at the back side of the antenna array at a specific distance. A prototype of the proposed antenna system is fabricated, and measurements are made to verify the simulated performance. From the results obtained, it is noted that the 1 × 4 planar antenna array with a metasurface reflector offers 12.86 GHz of impedance bandwidth in the range 27.14–40 GHz, and a maximum gain of 12.3 dBi is achieved in the operating frequency range. Furthermore, the directional radiation characteristics are obtained, especially for the low- and mid-band frequencies.

## Introduction

Recent developments in communication technology require systems that connect multiple devices to the wireless network at once, which can be fulfilled using frequency spectra that offer high bandwidth and high data rates. Federal Communications Commission (FCC) has therefore allocated a millimeter-wave (mm-wave) frequency spectrum to the fifth generation (5G) technology^1^. 5G technology offers extremely high data rates and improved connection reliability while consuming less power, even when many devices are connected simultaneously while providing wide range of advanced technologies as virtual and augmented reality (AR), smart automation, and Internet of Things (IoT) applications^2^. Because of these features, 5G has become an essential component of modern communication systems that demand fast, stable, and energy-efficient performance. To meet these requirements, devices designed for 5G communication are becoming smaller and more compact. These compact devices must accommodate various radio frequency (RF) components, including filters, amplifiers, and especially antennas, all within a limited space. As a result, antenna design has become a critical aspect of 5G device development. The antennas used in 5G systems must be compact while still providing wide bandwidth, high gain, and stable radiation performance to ensure reliable and high-speed communication. Among various antenna types, planar antennas are considered one of the best options due to their low profile, simple structure, lightweight design, and ease of integration with other electronic components on a printed circuit board. However, traditional planar antennas typically do not offer high gain, which limits their effectiveness in high-speed and long-range 5G communication systems.

One can enhance antenna gain by designing an array of radiating elements. In^3^, a modified inset-fed patch antenna array design was presented for 24 GHz radar applications. The designed array consists of 8 × 8 antenna elements, and they are fed using two feeding techniques, i.e., series feeding and corporate feeding. For both the arrangements, the arrays offered a peak gain of 25.9 and 25.2 dBi. In^4^, the authors designed an eight-element Vivaldi antenna array for 28 GHz applications. The array offered a maximum gain of 12.6 dBi in the operating range of 25–27.3 GHz. The authors in^5^ designed a circularly polarized high-gain slot array for 5G mm-wave applications. They designed an air-filled substrate-integrated waveguide (SIW)-based 1 × 8 slot antenna array on a three-layered printed circuit board (PCB) and one layer of metal, which employs a broadband feed network and the metal cavity radiating element, respectively. The designed multi-layered antenna array offers wideband response from 34.5 to 41.5 GHz and has a stable gain having a value of 17.5 dBi. Although these arrays offer high gain, they are large, and their usefulness in compact devices is limited.

Other techniques that can be used to enhance the antenna gain are to integrate metasurfaces, frequency selective surfaces (FSSs), or artificial magnetic conductors (AMCs) with the antenna or antenna array. In^6^, a high-gain metasurface-based patch antenna was designed for dual-band mm-wave applications. A simple inset-fed patch antenna was designed to get resonance around 26.5 GHz. The dual-band characteristics and enhanced gain were achieved by placing a 2 × 2 array of dual-layered metasurface structure above the antenna element. The incorporation of metasurface structure tends to achieve resonance at 26.45 and 27.62 GHz. Also, the gain of the antenna was enhanced by a value of 1.5 dB, where the peak value is observed to be 10 dBi for both operating frequencies. In^7^, a simple FSS-backed slot antenna was designed for 5G networks. To achieve the resonance at 28 GHz, a square slot was etched from the ground plane and excited with a 50Ω feed line. The improved radiation characteristics were achieved by placing an array of 5 × 5 FSS unit cells behind the antenna element. Through the designed configuration, the authors achieved a maximum gain of 10.3 dBi in the frequency band ranging from 25.2 to 31 GHz. In^8^, for gain enhancement, a phase gradient transmissive metasurface was designed and placed on the top of designed microstrip patch antenna. With the help of the designed metasurface, a 4.4 dB increase in the gain is observed where the maximum achievable gain is 12.4 dBi at 28 GHz. In^9^, the authors used an FSS array to enhance the gain of an SIW-based 1 × 8 slot antenna array. The array operates at 28.25 GHz and offers a peak gain of 12.42 dBi. In^10^, a 1 × 2 array of patch antennas was designed for wideband mm-wave applications. The gain of the array was enhanced with the use of a superstrate, which consists of 2 × 4 metamaterial unit cells. The presented configuration creates a Fabry-Perot cavity between the antenna array and superstrate, ultimately enhancing the antenna gain up to 12.7 dBi. They also presented a novel transmissive metasurface superstrate based compact patch antenna for 28 GHz mm-wave applications^11^. First, the authors improved the gain of the antenna by loading shorting pins on the radiating element and for further gain enhancement a metasurface superstrate was placed on top of the radiator. From the presented configuration, they achieved a maximum gain of 13 dBi in the frequency range of 26.62–29.79 GHz. In literature, some other techniques are also utilized for the enhancement of antenna gain. For example, in^12^, the authors designed a patch antenna array and enhanced its gain using two metal reflectors placed diagonally parallel to the array. The use of reflectors increased the gain up to 16.8 dBi. Despite high gain, the structure is bulky and cannot be used with handheld devices. The use of dielectric^13^ or meta-lenses^14^ is also feasible for antenna gain enhancement. The best use case of these kinds of configurations is their deployment in cellular base stations.

Overall observation from the presented literature is that the designed antennas or arrays provide high gain, they offer narrower bandwidth, some of them have bulky configurations, and they are large in size. Therefore, in this study, a low-profile, wideband metasurface-reflector-backed 1 × 4 planar antenna array is designed for mm-wave applications. The designed array has an overall physical size of 45 × 23.35 × 0.5 mm^3^ and electrical dimensions of 4.05 × 2.10 × 0.045 λ_0_^3^, where λ_0_ is the free space wavelength at 27 GHz. From the presented results, it is noted that the metasurface-backed antenna array resonates well from 27.14 to 40 GHz and offers a maximum gain of 12.3 dBi in the operating range. In addition, it exhibits a stable gain performance for mid- and high-band frequencies.

## Metasurface-backed antenna array

Before discussing the design and performance of the metasurface-backed antenna array, the design and performance parameters of the 1 × 4 antenna array and proposed metasurface structure are explained in detail.

### 1 × 4 planar antenna array

**Fig. 1:**
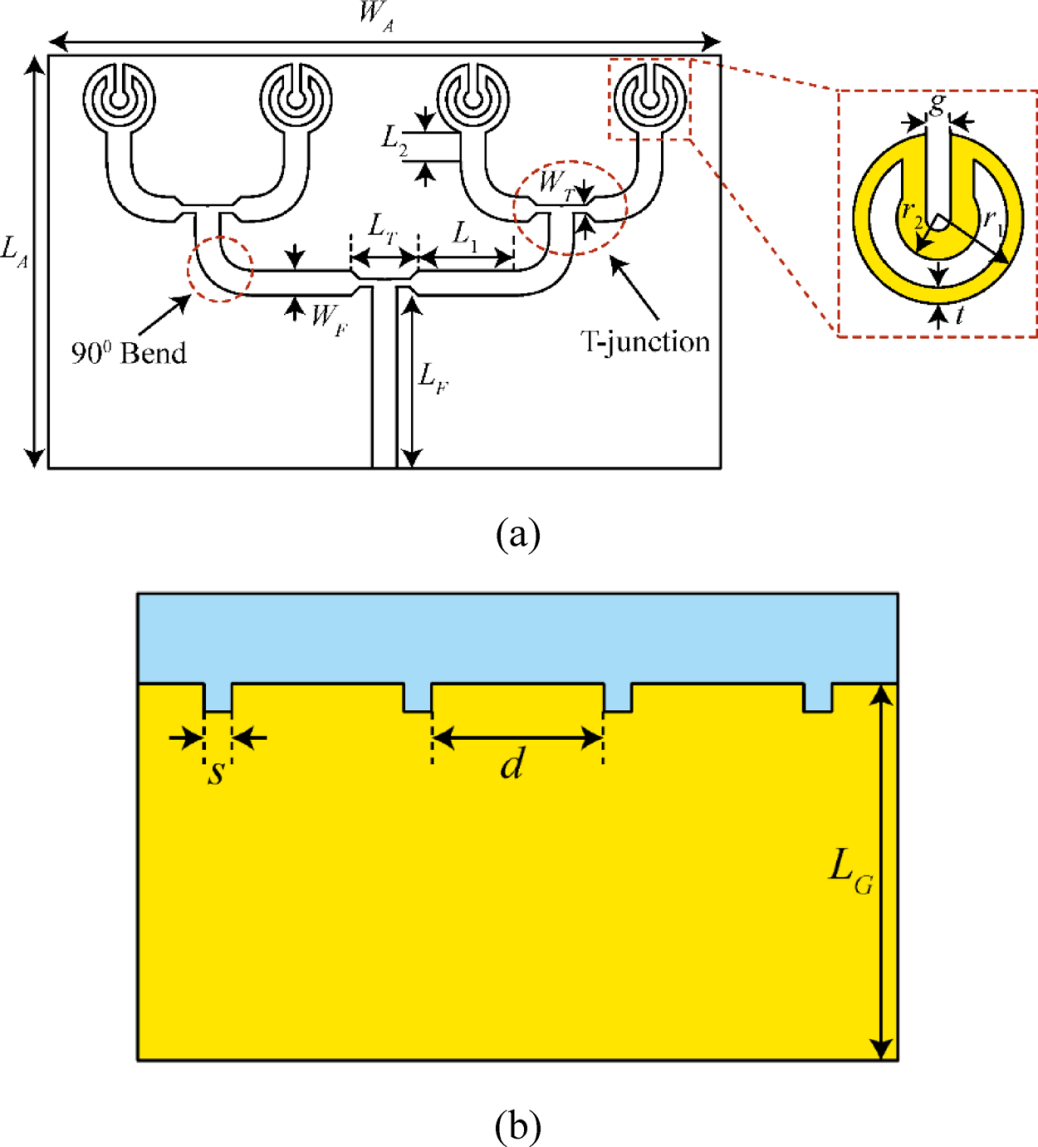
**a** Top- and **b** bottom-side of the 1 × 4 planar antenna array. The figure also shows the design of the broadband 1 × 4 corporate feeding network^15^. The dimensions are not to scale.

The schematic representation of the 1 × 4 planar antenna array is shown in Fig. 1. The array is designed on a low-loss Rogers RO-5880 dielectric substrate having relative permittivity (*ε*_*r*_) of 2.2, thickness of 0.51-mm, and tan δ of 0.0009. The single radiating element of the array is composed of a modified ring resonator, which is designed by combining two rings of different radii, as shown in Fig. 1(a). To feed the radiating elements, a broadband corporate feeding network is used, which consists of three different parts, i.e., microstrip lines, T-junctions, and 90^0^ bends. The purpose behind the use of a feeding network is to provide equal magnitude and phase of the signal input to each radiating element. A detailed description about the design and performance of the broadband corporate feeding network can be found in^15^. Moreover, to maintain high isolation among the radiating elements, the distance between them is taken to be 10 mm (> λ_0_/2). Also, to achieve a wideband response, a partial ground plane with a square notch is utilized, as shown in Fig. 1(b). The overall array parameters are as follows: *L*_*F*_ = 10, *W*_*F*_ = 1.4, *L*_*T*_ = 3.8, *L*_1_ = 5.4, *W*_T_ = 0.45, *L*_2_ = 1, *L*_*A*_ = 23.35, *W*_*A*_ = 38, *d* = 8.6, *s* = 1.4, *L*_*G*_ = 18.85, *g* = 0.5, *r*_1_ = 1.75, *r*_2_ = 0.75, *t* = 0.5 (all dimensions are in millimeters).

**Fig. 2:**
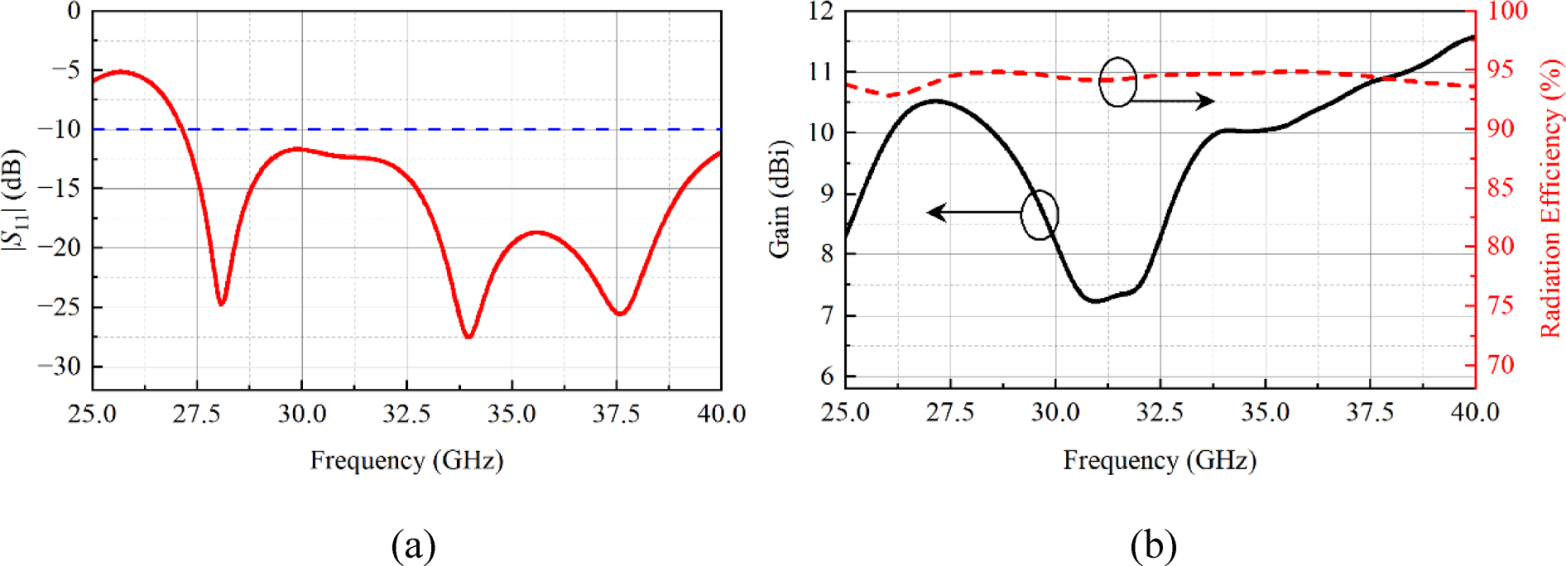
Simulated **a** |*S*_11_|, **b** radiation efficiency and gain of the planar antenna array.

The simulated reflection coefficient (|*S*_11_|) response is depicted in Fig. 2(a). The results show that the designed array resonates well from 27.14 to 40 GHz and has an impedance bandwidth of 12.86 GHz. Moreover, the radiation efficiency and gain of the array are shown in Fig. 2(b). The radiation efficiency is noted to be greater than 90% in the whole operating range. In addition, the gain of the designed array varies between 7.2 and 10.5 dBi, as shown in Fig. 2(b). From 29 to 33.5 GHz degradation in gain response is noted, which could possibly be associated with the impedance mismatch at these frequencies (see Fig. 2a).

**Fig. 3:**
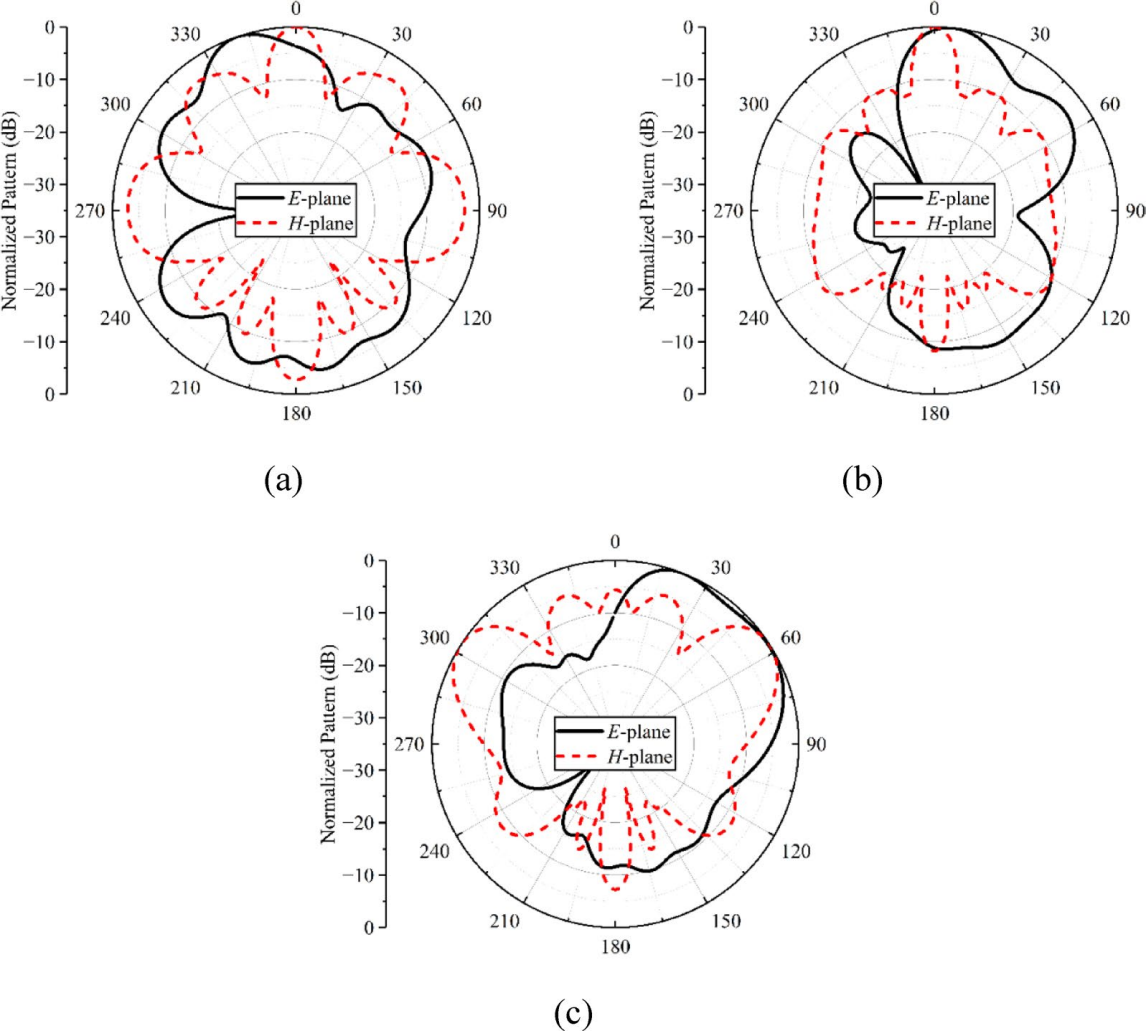
Simulated normalized radiation patterns of the planar antenna array at **a** 28 GHz, **b** 34 GHz, **c** 38 GHz.

The normalized far-field radiation properties of the antenna array for both the *E*- and *H*-planes are illustrated in Fig. 3. The radiation patterns are plotted for three frequency points, i.e., 28, 34, and 38 GHz. For 28 and 34 GHz, the *E*-plane pattern shows a typical monopole-like behavior (see Fig. 3a and b), while for 38 GHz, the *E*-plane pattern is directional where the main beam is directed towards 45^0^, as shown in Fig. 3(c). This could be associated with the unequal phase distribution of the designed broadband feeding network. In the *H*-plane, at 28 and 38 GHz, the array has quasi-omnidirectional radiation characteristics (see Fig. 3a and c). At 34 GHz, the pattern in the *H*-plane is directional, as shown in Fig. 3(b). One thing is also observed: that in the *H*-plane, the patterns are distorted, and these distortions may be associated with the excitation of higher-order modes.

To enhance the radiation characteristics of the proposed antenna, a metasurface reflector is introduced on the backside of the antenna array. This metasurface acts as a linear-to-linear polarization converter, significantly improving the antenna’s gain and radiation performance. By reflecting and reorienting the electromagnetic waves, it achieves better directivity and reduces backward radiation. The following subsections detail the design of the metasurface structure and its integration process with the planar antenna array.

### Metasurface reflector design

**Fig. 4:**
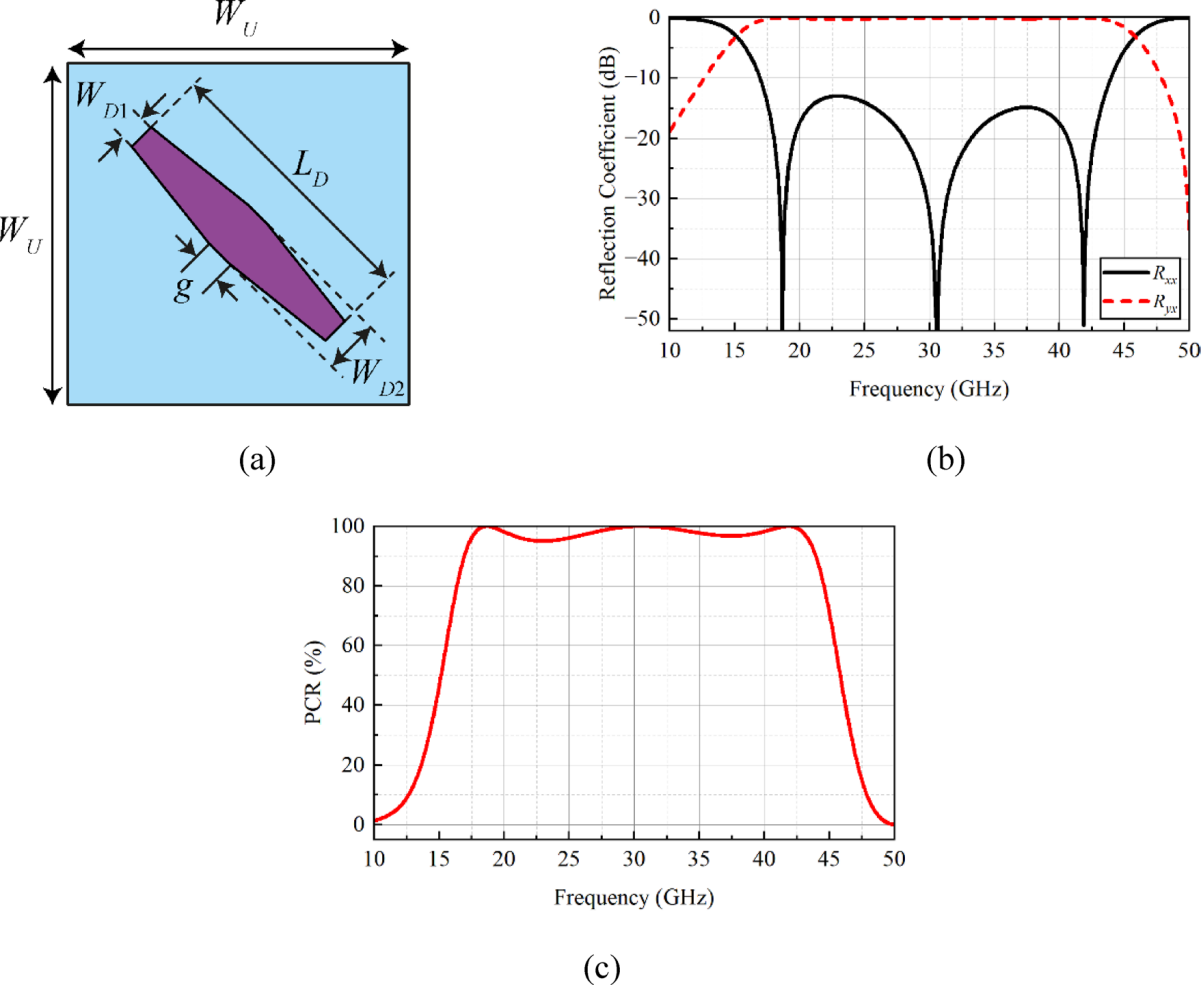
**a** Design, **b** reflection coefficient in terns of co- and cross-polarized components, and **c** PCR of the reflective metasurface structure. In (a), the dimensions are not to scale.

The unit cell design of the proposed reflective metasurface is depicted in Fig. 4(a). The unit cell is composed of a modified dipole resonator printed on the top side of a 1.57 mm thick low-loss Rogers RO-5880 dielectric with tan δ of 0.0009 and full ground plane. The overall design parameters of the metasurface unit cell are (all dimensions are in millimeters): *W*_*U*_ = 4.5, *L*_*D*_ = 5, *W*_*D*1_ = 0.5, *W*_*D*2_ = 1, and *g* = 0.5. To evaluate the performance of the metasurface, a commercially available electromagnetic (EM) solver, CST Microwave Studio, is used. In the simulation setup, unit cell boundary conditions are applied in the *x*- and *y*-axes, a Floquet port is assigned in the + *z*-axis, while an *E*_*t*_ = 0 boundary is applied in the -*z*-axis.

The co-polarized (*R*_*xx*_) and cross-polarized (*R*_*yx*_) reflection components of the metasurface structure are depicted in Fig. 4(b). Mathematically, they can be evaluated as^16^:

1 $$\:{R}_{xx}=\frac{{E}_{x}^{r}}{{E}_{x}^{i}}$$

2 $$\:{R}_{yx}=\frac{{E}_{y}^{r}}{{E}_{x}^{i}}$$

where $\:{E}_{x}^{r}$ and $\:{E}_{y}^{r}$ represent the reflected electric fields of the *x*- and *y*-polarized waves, respectively, while $\:{E}_{x}^{i}$ denotes the incident electric field of the *x*-polarized wave. From Fig. 4(b), one can observe the metasurface operates well from 15.2 to 45.88 GHz, and it well covers the operating frequency range of the designed antenna array. In addition to reflection properties, the second important parameter that needs to be evaluated is the polarization conversion ratio (PCR), which can be calculated as described in^16^.

3 $$\:\mathrm{P}\mathrm{C}\mathrm{R}=\frac{{\left|{R}_{yx}\right|}^{2}}{{\left|{R}_{yx}\right|}^{2}+{\left|{R}_{xx}\right|}^{2}}$$

For the designed metasurface, the value of PCR is noted to be greater than 90% for the entire operational bandwidth, as shown in Fig. 4(c). This shows that the metasurface can effectively convert the incident wave polarization from one state to another.

### Integration of antenna array and metasurface

**Fig. 5:**
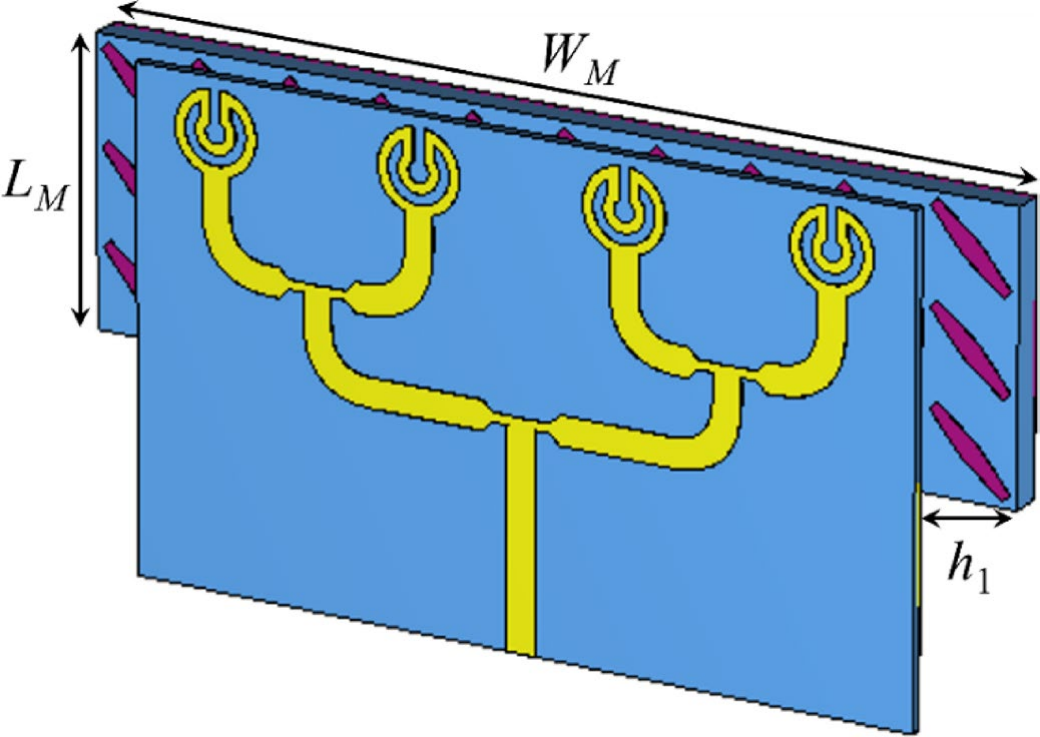
Configuration of a metasurface reflector-based planar antenna array. The dimensions are not to scale.

After evaluating the performance of the metasurface unit cell, an array of the same structure is designed (see Fig. 5) and integrated with the antenna array to achieve high gain. The metasurface array has overall dimensions of 45 × 13.5 mm^2^. The final configuration of the metasurface-backed antenna array is depicted in Fig. 5. Note that the metasurface array is placed on the back side of the radiating elements so that it reflects the backward radiations in the broadside direction and improves the radiation properties. A detailed theoretical background on how a reflector-based antenna system works is provided below.

While at front of the reflector, the radiating structure (in our case array) is put, the radiation response of the proposed structure becomes broadsided as backside lobes are reflected. Hence, when the two wave components are in phase, they reinforce each other through constructive interference, which directs the radiated energy toward the broadside direction and enhances the antenna’s overall gain. Consider that *ϕ*_*T*_ and *ϕ*_*R*_ are the transmitted and reflected wave phases, and the complete propagation trip between reflector can be denoted by *ϕ*_*S*_. Mathematically, the relationship between these phases can be expressed as^17^:

4 $$\:{\varphi\:}_{T}={\varphi\:}_{R}+{\varphi\:}_{S}$$

where

5 $$\:{\varphi\:}_{S}=4\pi\:f\times\:\frac{{h}_{1}}{c}$$

where c is light speed in vacuum, h1 is the gap between the reflector and the radiator, and *ϕ*_*T*_ should be the integral of 2π or zero^17^. To evaluate the performance of the design array system, initially, a parametric study was conducted where the air gap, denoted by *h*_1_, between the radiating array and the metasurface reflector, based on 3 × 10 unit cells, was changed. This gap ensures the constructive interference of radiation and reflected waves, and its value must be an integer multiple of λ_0_. As the metasurface offers wideband performance, the air gap can be optimized to get acceptable radiation performance^17^.

**Fig. 6:**
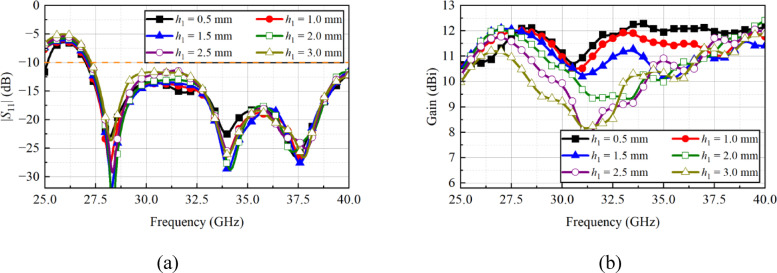
Simulated **a** |*S*_11_|, and **b** gain of the antenna array when the value of *h*_1_ is changed from 0.5 to 3.0 mm.

The study was conducted by changing the value of parameter *h*_1_ from 0.5 to 3 mm with a step of 0.5 mm. The change in the *h*_1_ value has no effect on the |*S*_11_| response (see Fig. 6a), but it has a significant effect on the gain response of the array. As the value of *h*_1_ increased from 0.5 to 3.0 mm, the gain dropped in the operating range, as shown in Fig. 6(b). Therefore, based on the results, *h*_1_ = 0.5 mm is chosen as the final design parameter, where the gain varies in the range of 11.5 to 12 dBi, and it is almost constant after 32 GHz.

**Fig. 7:**
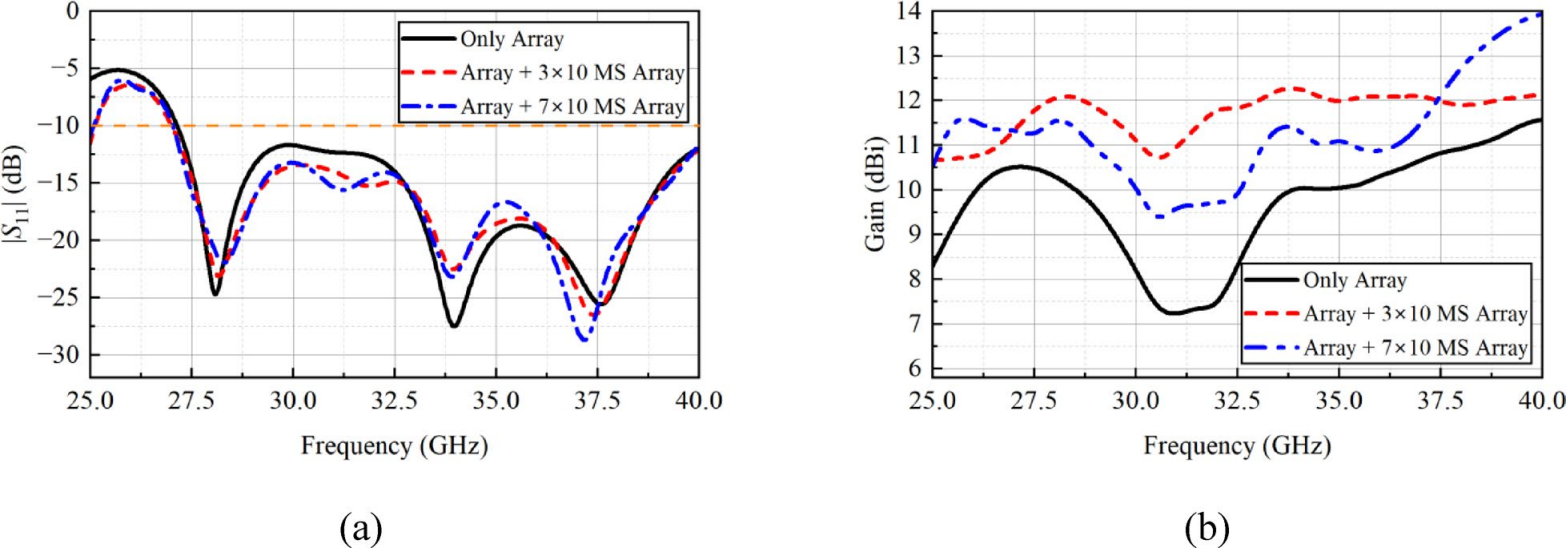
Simulated **a** |*S*_11_|, and **b** array gain for different metasurface configurations.

After finalizing the *h*_1_ value, another study was conducted where the array performance was assessed in the presence of different metasurface arrays, where the arrays consist of 3 × 10 and 7 × 10 unit cells. One can observe from Fig. 7(a) that different metasurface arrays have no effect on the |*S*_11_| characteristics. On the other hand, the metasurface array with 7 × 10 unit cells have less gain compared to an array composed of 3 × 10 unit cells, as shown in Fig. 7(b). Based on the study, for the final design, a metasurface array that consists of 3 × 10 unit cells are chosen. Moreover, with the use of a metasurface reflector, the gain of the designed planar array is increased with an overall average value of 2.5 dB. For the low-, mid-, and high-band frequencies, the gain is increased by a value that lies in the range of 1–4 dB.

## Fabrication and measured performance

**Fig. 8:**
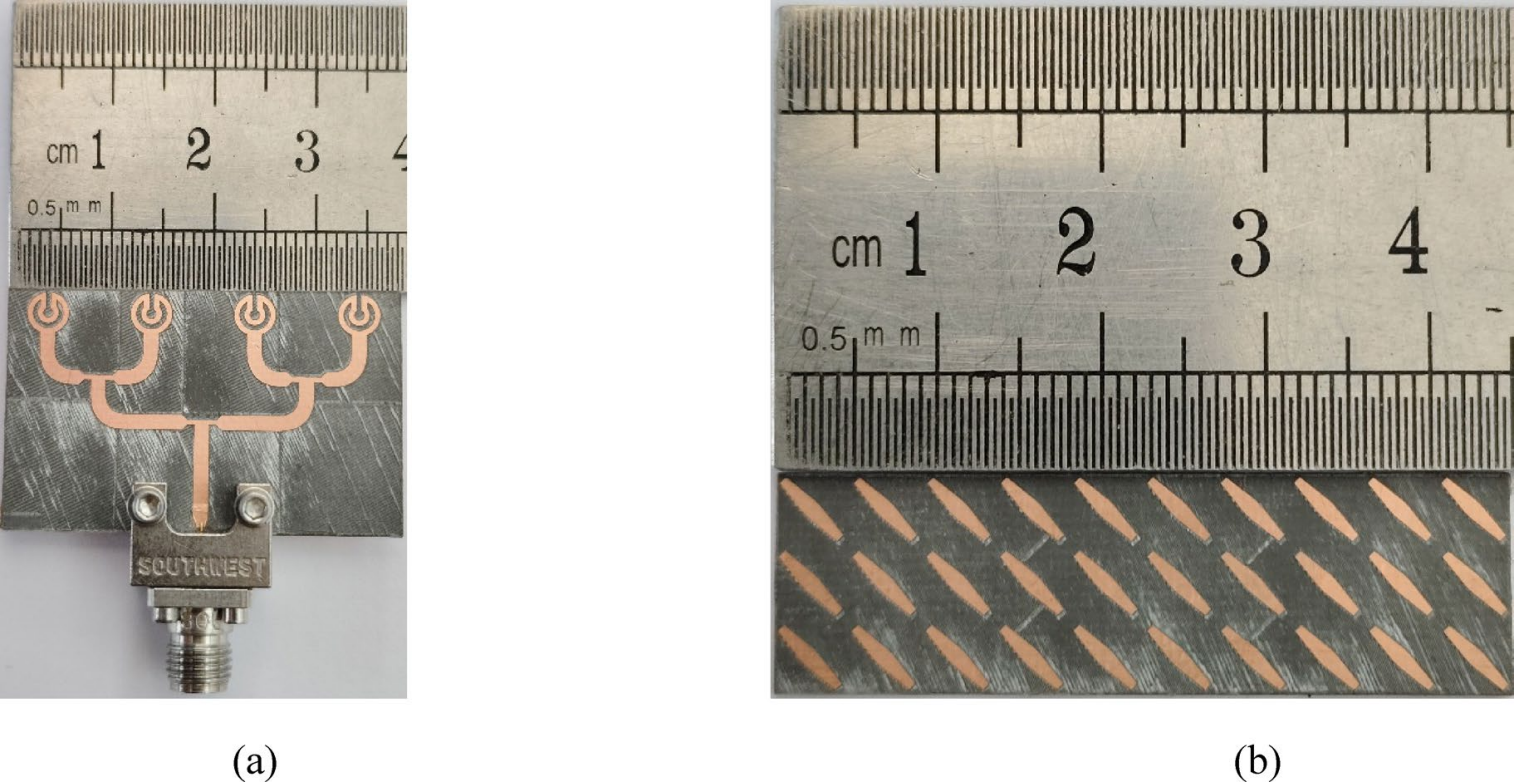
Prototype of the **a** planar antenna array and **b** 3 × 10 metasurface array.

**Fig. 9:**
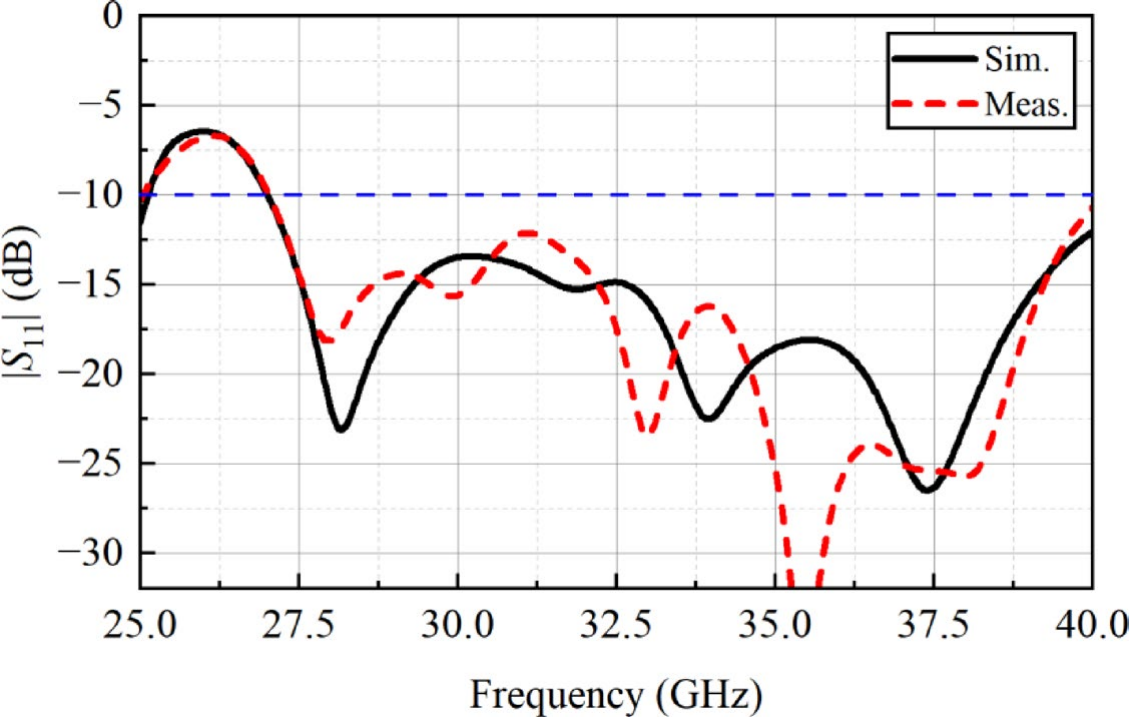
*S*_11_| of metasurface-backed planar antenna array.

For verification of simulation, a prototype of the proposed metasurface based planar antenna array was fabricated (see Fig. 8). Both the planar antenna array and metasurface array were fabricated separately, and then they were combined using a foam whose permittivity is close to air. For |*S*_11_| characterization, a precision network analyzer (PNA) was used where a one-port calibration was done in the frequency range of 25–40 GHz. The simulated and measured |*S*_11_| of the proposed array is shown in Fig. 9, and it is observed that designed array system resonates well in the desired frequency range, i.e., 27.14–40 GHz. A minor discrepancy in the results may arise due to connector and RF cable losses, and fabrication tolerances.

**Fig. 10:**
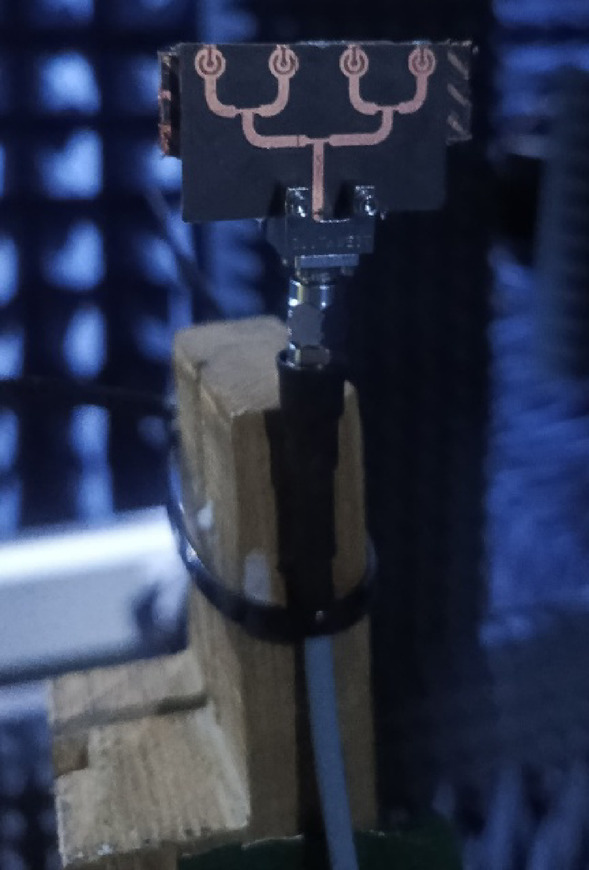
Measurement setup for far-field characterization.

**Fig. 11:**
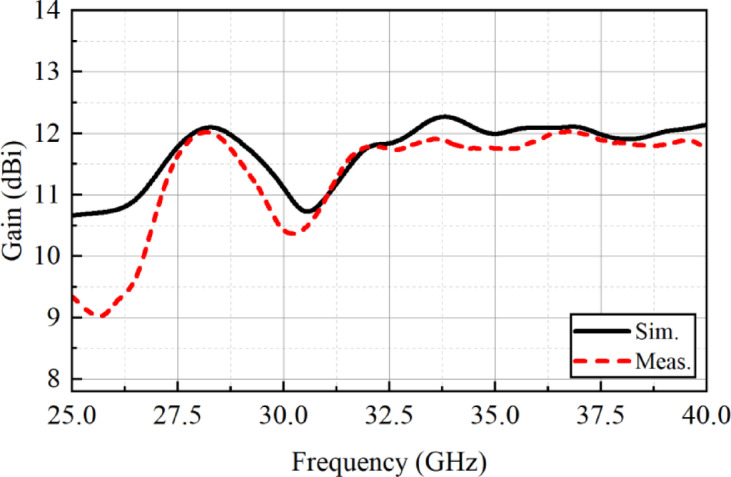
Gain of the metasurface-backed planar antenna array.

For far-field measurement, the fabricated antenna array was tested in an anechoic chamber following a standard measurement procedure, as shown in Fig. 10. During the experiment, the metasurface-backed planar antenna array was mounted on a rotatable turntable and positioned at an appropriate distance from a reference standard gain horn antenna to ensure accurate far-field measurements. Figure 11 presents a comparison between the simulated and measured gain responses of the proposed array. The data indicates that the measured gain closely matches the simulated results, confirming a strong correlation between simulation and practical performance. The average measured gain of the antenna array is approximately 9 dBi, which aligns well with the simulated value. Additionally, the maximum simulated and measured gain values are recorded as 12.3 dBi and 12 dBi, respectively. This suggests that the antenna maintains high radiation efficiency and stable gain characteristics under real-world testing conditions.

**Fig. 12:**
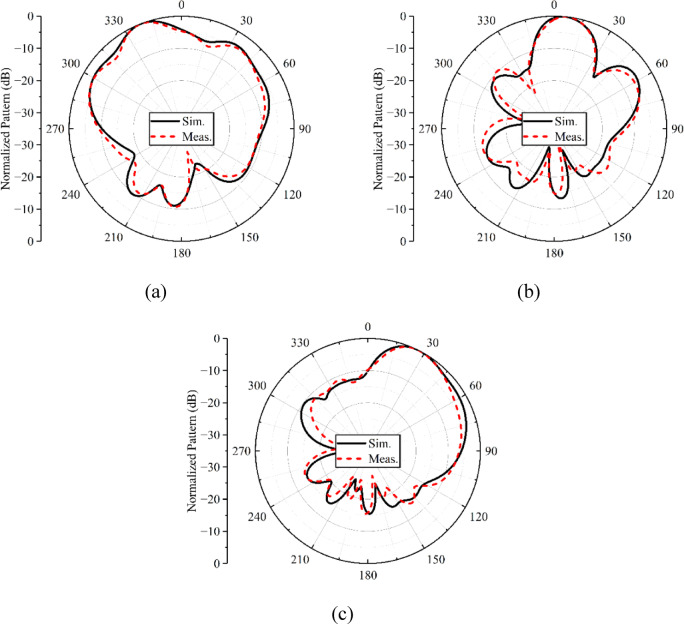
Normalized E-plane radiation patterns of the metasurface-backed planar antenna array at **a** 28 GHz, **b** 34 GHz, **c** 38 GHz.

**Fig. 13:**
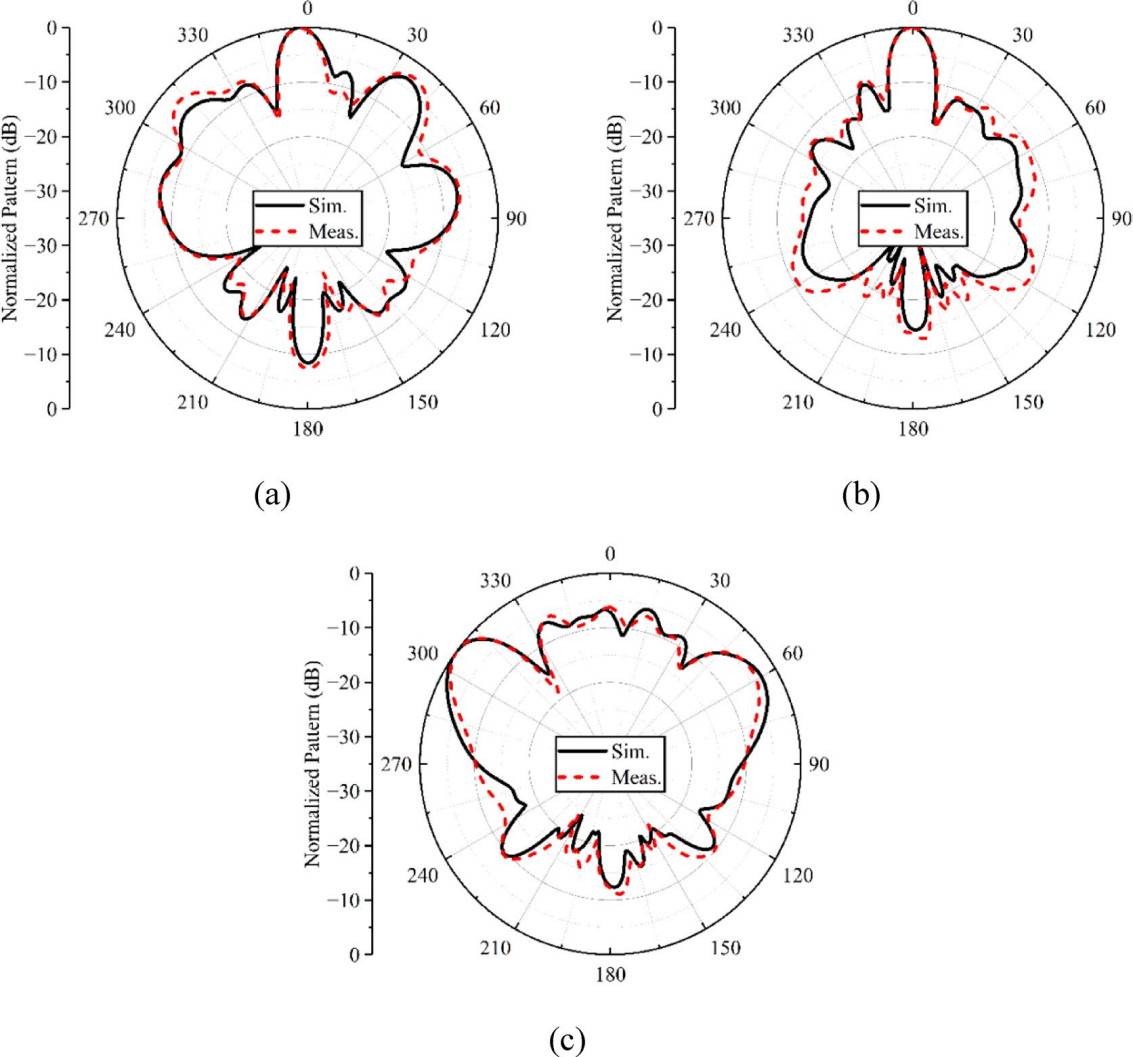
Normalized H-plane radiation patterns of the metasurface-backed planar antenna array at **a** 28 GHz, **b** 34 GHz, **c** 38 GHz.

The simulated and measured normalized far-field radiation patterns of the proposed metasurface-backed planar antenna array for both *E*- and *H*-planes are shown in Figs. 12 and 13. The patterns are plotted for three distinct frequency points, i.e., 28, 34, and 38 GHz. With the use of a metasurface reflector at the back side of the array, the patterns in both the planes become directional, as shown in Figs. 12(a, b) and 13(a, b). At 38 GHz (see Figs. 12c and 13c), the metasurface has no effect on the radiation patterns in both planes except increasing the gain (see Fig. 11). Although the metasurface reflector offers directional radiation characteristics, shifting the beam direction to certain angles. The observed beam squint across the operating frequency band is primarily attributed to the frequency-dependent phase response of the metasurface reflector and the inherent dispersive behavior of the planar antenna array. While the metasurface provides phase compensation around the design frequency, the reflection phase varies with frequency, leading to slight misalignment of the reflected fields at frequencies away from the center. Additionally, the array element spacing contributes to phase sensitivity, which further accentuates beam deviation at band edges. Despite this frequency-dependent beam shift, the squint remains within acceptable limits, especially for low- and mid-band frequencies, i.e., 28 and 34 GHz. The beam squint can be mitigated by employing multi-resonant or tunable metasurface elements. One can also improve the radiation properties of the antenna with the help of metasurface-based slot antenna designs^18,19^, which will be the focus of future research activities.

From the above presented results, shown in Figs. 11, 12 and 13, it is noted that the proposed design provides a modest gain improvement of approximately 2.5 dB compared to the antenna without the metasurface reflector. While the enhancement in gain is limited, the primary motivation for incorporating the metasurface is to achieve broadside radiation patterns, maintaining polarization purity, and suppressing surface-wave interactions. These features are particularly valuable in mm-wave applications, where planar designs with low profile are preferred. The modest gain improvement should thus be considered in the context of the overall design objectives.

Table 1 presents a comparison between the proposed and previously presented state-of-the-art reflector-based antenna designs. Although the designed array is large in size, it offers wide impedance bandwidth compared to the designs presented in^6,7,9–11^ and offers comparable gain as per the designs reported in^9,10^. In addition, the overall thickness of the proposed metasurface reflector-based array is compact compared to the designs presented in^6,7,9–11^.

**Table 1:** Comparsion between proposed and previously presented state-of-the-art reflector based antennas.

Ref.	Antenna Size		Antenna Type	Overall Thickness (mm)	Frequency (GHz)	Bandwidth (GHz)	Gain (dBi)	Reflector Type
	mm^2^	λ^2^						
^6^	21 × 20	1.82 × 1.73	Single Patch	3.11	26.4/27.4	1/0.8	10/9.6	Transmissive Metasurface
^7^	25 × 25	2.12 × 2.12	Slot Antenna	5.00	25.5–30.8	5.3	10.3	Reflective FSS
^9^	68.42 × 10.06	6.44 × 0.94	Slotted Array	5.70	28.24	0.15	12.41	Reflective FSS
^10^	20.53 × 20.63	1.84 × 1.85	1 × 2 Array	6.34	27.38–33.34	5.96	12.7	Transmissive Metasurface
^11^	14.50 × 18.00	1.35 × 1.68	Single Patch	6.22	26.62–29.79	3.17	13	Transmissive Metasurface
***Prop.***	***45 × 23.35***	***4.05 × 2.10***	***1 × 4 Array***	***2.58***	***27.14–40.14***	***12.86***	***12.3***	***Reflective*** ***Metasurface***

## Conclusions

A metasurface reflector-based planar antenna array is designed for wideband 5G mm-wave applications. The proposed design is low-profile and compact so that it can be easily integrated into handheld devices. For the antenna array design, modified ring resonators are used, fed using a broadband corporate feeding network. For wideband response, a partial ground plane with a square notch is used. To enhance the radiation performance of a designed antenna array, a metasurface reflector based on modified dipole elements is designed and placed behind the antenna array. It is observed that the designed antenna array operates well in the mm-wave frequency range from 27.14 to 40 GHz and exhibits a peak gain of 12.3 dBi in the operating bandwidth. Moreover, the designed metasurface-reflector-backed antenna array offers stable gain performance for high-band frequencies in the range of 32–40 GHz. Additionally, the simulation and measurement performance of the antenna array agreed with each other very well.

## Data Availability

All data generated or analyzed during this study are included in this article.
